# Land Use Land Cover Changes in Detection of Water Quality: A Study Based on Remote Sensing and Multivariate Statistics

**DOI:** 10.1155/2017/7515130

**Published:** 2017-03-09

**Authors:** Ang Kean Hua

**Affiliations:** Department of Environmental Sciences, Faculty of Environmental Studies, Universiti Putra Malaysia (UPM), 43400 Serdang, Selangor, Malaysia

## Abstract

Malacca River water quality is affected due to rapid urbanization development. The present study applied LULC changes towards water quality detection in Malacca River. The method uses LULC, PCA, CCA, HCA, NHCA, and ANOVA. PCA confirmed DS, EC, salinity, turbidity, TSS, DO, BOD, COD, As, Hg, Zn, Fe,* E. coli*, and total coliform. CCA confirmed 14 variables into two variates; first variate involves residential and industrial activities; and second variate involves agriculture, sewage treatment plant, and animal husbandry. HCA and NHCA emphasize that cluster 1 occurs in urban area with Hg, Fe, total coliform, and DO pollution; cluster 3 occurs in suburban area with salinity, EC, and DS; and cluster 2 occurs in rural area with salinity and EC. ANOVA between LULC and water quality data indicates that built-up area significantly polluted the water quality through* E. coli*, total coliform, EC, BOD, COD, TSS, Hg, Zn, and Fe, while agriculture activities cause EC, TSS, salinity,* E. coli*, total coliform, arsenic, and iron pollution; and open space causes contamination of turbidity, salinity, EC, and TSS. Research finding provided useful information in identifying pollution sources and understanding LULC with river water quality as references to policy maker for proper management of Land Use area.

## 1. Introduction

Land Use Land Cover (LULC) refers to two separate terminologies that are often used interchangeably [1, 2]. Land Cover can be defined as the physical characteristics of the earth's surface which involve vegetation, water, soil, and other physical features created through human activities like settlements, while Land Use refers to land used by humans for habitats concerning economic activities [1]. LULC patterns depend on human usage in terms of natural and socioeconomic development through space and time. In other words, Land Use changes have the ability to affect the Land Cover and vice versa. Shifting into possibility negative impact through the Land Use perspective for social activities is affecting Land Cover to change, especially in biodiversity, water and earth radiation, trace gas emission, and other processes that come together to affect the climate and biosphere [2, 3]. These changes are attributed to only one main factor in terms of size and pattern, namely, “population growth.” Increasing population growth directly and indirectly contributes to LULC changes, especially from the perspective of demand for built-up area, agricultural activities, and water resources. Ecological expertise is very concerned with LULC changes that impact biodiversity and aquatic ecosystems [4]. LULC changes in a watershed will affect water quality, leading to increased surface runoff, reduced groundwater discharge, and transfer of pollutants [2, 4]. Therefore, LULC information at the watershed level is important for selection, planning, monitoring, and management of water resource so that the changes in Land Use meet the increasing demand for human needs and welfare without compromising water quality.

Various research studies have been conducted about the change analysis of watersheds, which are important in developing effective management strategies to protect water resources [1, 5–7]. Watershed management is necessary because a watershed is not only a hydrological unit [8] but also plays an important part in socioecological perspective by providing economical, food, and social security as well as provision of life support services to local residents [9]. LULC changes in the watershed area for urbanization and deforestation will continuously have negative impacts on water quality and indirectly affect the nature of a watershed ecosystem. Hence, understanding of the spatial and temporal variations that occur in a watershed over time as well as explanation of the interaction between hydrological components of the watershed will allow better water conservation strategies to be formulated [5]. Specifically, remote sensing has been widely used to classify and map LULC changes with different techniques and data sets, such as Landsat images that provide better classification of different landscape components at a large scale [10]. Several change detection techniques have been developed in remotely sensed image with continuous debate on the advantages and disadvantages of each technique. These include unsupervised classification or clustering, supervised classification, PCA, hybrid classification, and fuzzy classification, which are all commonly applied and used in classification [1, 2, 11, 12]. Although various classification techniques have been proposed, supervised classification methods are considered as favorable for change detection analysis. More recently, researchers have applied supervised classification for several LULC change detection for several research aims and purpose [1–3, 13].

The Malacca River watershed area has been selected for a change detection study because of its uncontrolled urbanization, unmanageable sewage discharge, and active soil erosion and tree cutting. Apart from these actions, pesticide residues and animal husbandry residues are suspected to become major concerns in the watershed area due to increasing agricultural and poultry farm activities [14]. Rapid urban development in the study area has led to several problems like fragmentation of aquatic animals, soil erosion, and river pollution due to deforestation and discharge of municipal garbage and industrial waste [15]. This study is carried out using the remote sensing application to differentiate the extent of changes which occurred in the Malacca River watershed for 15 years. The objectives of this study are to examine the potential sources of pollutants in the Malacca River between 2001, 2009, and 2015; identify the different LULC classes and the pattern of changes in watershed from 2001 to 2009 and 2009 to 2015; and determine the connection of LULC changes in contributing to pollutant sources in the Malacca River.

## 2. Materials and Methods

### 2.1. Study Area

**Figure 1:**
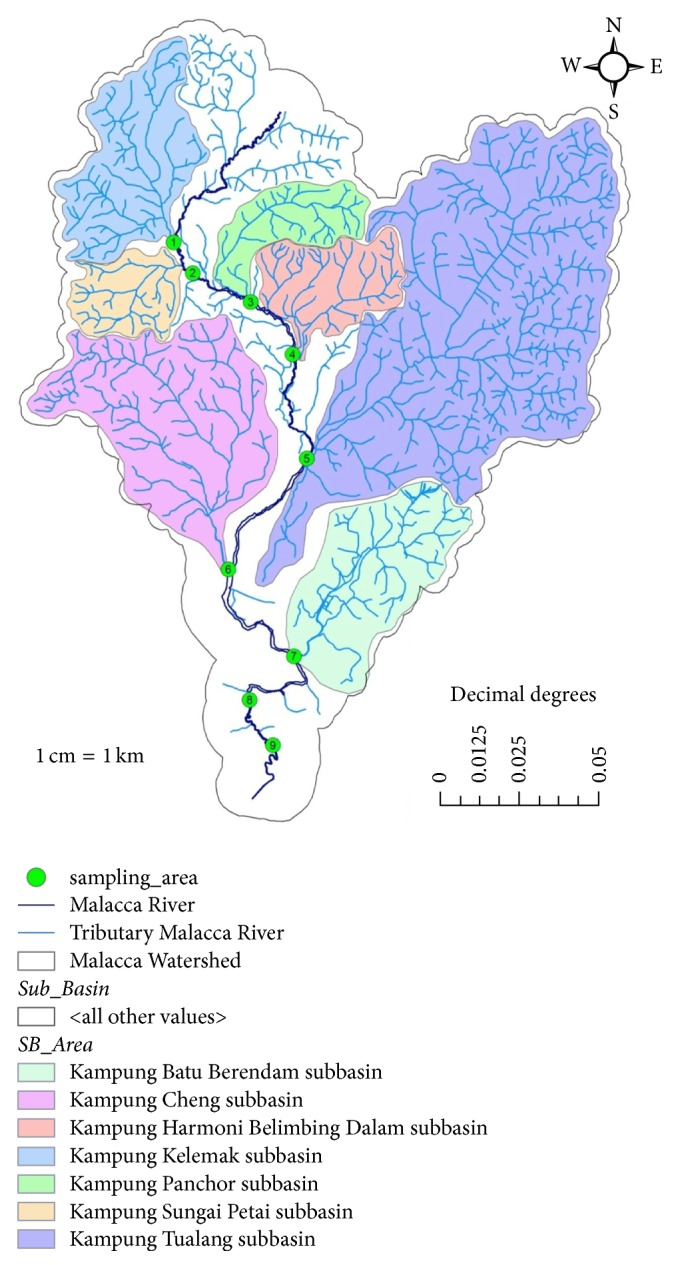
Sampling stations and subbasin of the study area.

Malacca state is located in the South West of Peninsular Malaysia. The geographical coordinates are 2°23′16.08′′N to 2°24′52.27′′N for latitude and 102°10′36.45′′E to 102°29′17.68′′E for longitude. Malacca state can be divided into three districts, namely, Alor Gajah, Jasin, and Malacca Central. The catchment areas in Malacca state are approximately 670 km^2^ and contain an 80 km length of the Malacca River that flows through Alor Gajah and Malacca Central. Generally, the river is formed by 13 subbasins of watershed, namely, Kampung Ampang Batu Gadek subbasin, Kampung Balai subbasin, Kampung Batu Berendam subbasin, Kampung Buloh China subbasin, Kampung Cheng subbasin, Kampung Gadek subbasin, Kampung Harmoni Belimbing Dalam subbasin, Kampung Kelemak subbasin, Kampung Panchor subbasin, Kampung Pulau subbasin, Kampung Sungai Petai subbasin, Kampung Tamah Merah subbasin, and Kampung Tualang subbasin. Only 7 subbasins of 13 were selected, with 9 sampling stations along the river (Figure 1).

Malacca state has a reservoir located between Alor Gajah and Malacca Central. This is the Durian Tunggal Reservoir, with a catchment of 20 km^2^. It acts as a source of water for Malacca residents. Increasing local population has led to increasing public facilities such as transport, healthcare, accommodation, sewage, and water supply services [14–16]. Due to the drastic population growth, rapid urban development in the Strait of Malacca has also increased, especially from a Land Use perspective. A majority of residents are centralized in the city, which extends about 10 km to the west, 10 km to the east, and 20 km to the north. The changes in Land Use have continuously developed until today, which is in line with the vision and mission of sustainable tourism sector. Eventually, these actions indirectly contribute to economic growth and political changes, strengthened cultural and social relationships, and also impact environmental quality, especially the water in Malacca River.

## 3. Data Collection

Nine sampling stations were chosen along the Malacca River. River water quality data included samples in year 2015 analyzed based on APHA [17], while river water quality data for 2001 and 2009 were collected from the Department of Environment (DOE), Malaysia. The primary data was collected in 2015 to obtain recent water quality data status as well as field data verification. There are only two methods of measurements involved: in situ analysis and laboratory analysis. River water quality was analyzed according to physicochemical parameters, that is, pH, temperature, electrical conductivity (EC), salinity, turbidity, total suspended solid (TSS), dissolved solids (DS), dissolved oxygen (DO), biological oxygen demand (BOD), chemical oxygen demand (COD), and ammoniacal nitrogen (NH_3_N), trace elements (i.e., mercury, cadmium, chromium, arsenic, zinc, lead, and iron), and biological parameters (i.e.,* Escherichia* coliform and total coliform) as shown in Table 5. Additionally, the remote sensing imagery of selected research areas for 2001, 2009, and 2015 was obtained from ARSM and downloaded from the USGS Earth Explorer of the selected research area in Malacca state. Landsat 5 TM data were obtained for 2001 and 2009, while Landsat 8 data was obtained for 2015.

## 4. Data Analysis

### 4.1. River Water Data

#### 4.1.1. Water Quality Analysis

Water samples were analyzed based on in situ measurement and laboratory analysis. In situ measurement involves pH testing using a SevenGo Duo pro probe (Mettler Toledo AG); turbidity test using a portable turbidity meter (Handled Turbidimeter Hach 2100); and multiparameter probe (Orion Star Series Portable Meter) tests on temperature, EC, DS, salinity, and DO. Meanwhile, laboratory analysis involves measurement on NH_3_N using a spectrophotometer based Hach Method 8038; COD parameter using APHA 5220B open reflux technique; BOD parameter measure using APHA 5210B (Hach Method 8043); TSS measure using APHA 2540D method;* E. coli* and coliform test using membrane filtration method based APHA 9221B; and trace metal test using an inductive coupled plasma-mass spectrometry (ICP-MS, ELAN DRC-e, Perkin Elmer). Each sample underwent the tests in triplicate before calculating the mean value, and standard deviation (SD) was used as an indication of the precision of each parameter measured with less than 20%.

#### 4.1.2. Statistical Analysis

The analysis results are then input into Statistical Package for Social Science (SPSS) version 23 for statistical analysis using principal component analysis (PCA), canonical correlation analysis (CCA), hierarchical cluster analysis (HCA) and nonhierarchical cluster analysis (NHCA), and analysis of variance (ANOVA). Generally, PCA can be expressed through (1) original data reduced to dominant components of factors (source of variation) that influence the observed data variance and (2) the whole data set extracted to produce eigenvalue and eigenvectors [18]. Only eigenvalues greater than 1 are considered significant [19] to perform new group variable Varimax Factor (VFs). A VFs coefficient with 0.6 is considered “moderate” and will be taken into account as factor loadings. PCA is applied in this study to define possibility of pollutant sources in the Malacca River. Continuously, the components of PCA will be extracted into CCA for further analysis. CCA have an ability to investigate relationship between the two groups. In other words, (1) CCA will seek for vectors of a and b in random variables of *αX* and *βY* to maximize the correlation of *ρ* = corr(*αX*, *βY*); (2) random variable of *U* = *αX* and *V* = *βY* will be constructed to perform new sets of canonical variates that are linear combinations from the original variables with simple correlation between *U* and* V*; (3) then other vectors *U* and *V* having maximal correlation subject but being uncorrelated with the first canonical variate will be produced as the second canonical variates [20]. CCA is applied in this study to determine accurately and precisely pollutant sources in the river. HCA is able to sort different objects into the same group based on similarity between objects, which involve (1) Ward's methods using variance analysis to minimize between any two clusters [18, 21]; (2) measuring the similarity through Euclidean distance between two samples [18, 21]; and (3) a dendogram to provide the results for high similarity with small distances between clusters in a group [12]. This study employed HCA to determine possible area contributing to pollution in the study area. Unlike HCA, NHCA with the involvement of *K*-means method is used to obtain the correct classification of pollutant sources based on the PCA components provided. Lastly, ANOVA will be used to analyze between Land Use classes of LULC changes analysis with water quality from factor loadings of PCA analysis. The main purposes of using ANOVA are to determine and to prove the existing of LULC classes that react as pollutant sources to impact the water quality and cause contamination in the Malacca River.

### 4.2. Remote Sensing Data

#### 4.2.1. Image Preprocessing, LULC Classification, and Change Detection Analysis

Satellite images required preprocessing to ensure that the primary object could be established into a more direct affiliation between acquired data and biophysical phenomena [1]. The preprocessing was accomplished using ArcGIS version 10.0 for georeferencing, mosaicking, and subsetting of the image for the Area of Interest (AOI). Landsat 8 images underwent spatial sharpening using the panchromatic bands which resulted in images with a 15 m resolution. Meanwhile, Landsat 5 TM images for 2001 and 2009 were in an original 30 m resolution. Further image processing analysis was carried out using ENVI 5.0. The image was displayed in natural color composite using a band combination of 3, 2, and 1 for Landsat 5 TM and 4, 3, and 2 for Landsat 8. Maximum likelihood supervised classification was performed using several selected regions, with Regions of Interest (ROIs) based on delineated classes of agriculture, built-up areas, water, and open space area (Table 1).

In performing LULC change detection, a postclassification detection method is applied in ENVI 5.0, which involves two independently classified images used to make comparisons to produce change information on a pixel basis. The interpretation between images provides changes in “-from, -to” information. Classified images of two different data sets were compared using cross-tabulation in determining qualitative and quantitative aspects of changes for the periods from 2001 to 2009 and 2009 to 2015. The magnitude of change and percentage of changes can be expressed in a simple formula as follows:

(1) K=F−I,A=F−II×100,

where *K* is magnitude of changes, *A* is percentage of changes, *F* is first data, and *I* is reference data [11].

#### 4.2.2. Accuracy Assessment

Accuracy classification assessments for 2001, 2009, and 2015 images were carried out to determine the quality of information provided from the data. If classification data is to be used for change detection analysis, it is important to conduct accuracy assessments for individual classifications [1]. Kappa test is used to perform measurement of the classification accuracy as the test is able to account for all elements in confusion matrix including diagonal elements [22]. A kappa test is a measure calculated using predefined producer and user assigned ratings, which can be expressed as follows:

(2) $$\begin{array}{c} K=\frac{P\left(A\right)-P\left(E\right)}{1-P\left(E\right)}, \end{array}$$

where *P*(*A*) is the number of times the *k* raters agree and *P*(*E*) is the number of times the *k* raters are expected to agree only by chance [1, 23]. Meanwhile, user accuracy can be defined as the probability that a pixel in an image actually represents a class on the ground, while producer's accuracy indicates the probability a pixel being correctly classified and is mainly used to determine how well an area can be classified [23]. As described previously, the four categories of classes that have been delineated should have a minimum of 50 points for each considered category to increase the percentage of accuracy assessment [1]. Therefore, this study indicates the overall classification accuracies for 2001, 2009, and 2015 are 89.51%, 88.49%, and 92.21%, with kappa statistics of 0.87, 0.85, and 0.90, respectively. According to Weng [24], the minimum level for accuracy assessment in identification of Land Use and LULC classes in remote sensing data should be at least 85%.

## 5. Results and Discussions

### 5.1. Magnitude and Percentage of LULC Changes between 2001–2009 and 2009–2015

The magnitude and percentage of LULC changes from 2001 to 2009 and 2009 to 2015 are summarized in Table 2. The results indicate Land Use type in 2001 for built-up area is 196 km^2^ (29.3%), agriculture is 271 km^2^ (40.4%), water is 138 km^2^ (20.6%), and open space is 65 km^2^ (9.7%). In 2009, only built-up area and open space had increased, by about 49 km^2^ (7.3%) and 61 km^2^ (9.1%) to become the total of 245 km^2^ (36.6%) and 126 km^2^ (18.8%), respectively. However, agriculture and water are reduced by about 10.3% and 6.1%, which resulted in the total area of 202 km^2^ and 97 km^2^. Lastly, built-up areas have continuously increased by about 13.7% to provide a total area of 337 km^2^, and agricultural land also increases for 2.9% to perform total area of 221 km^2^. Nevertheless, open space areas have decreased about 44 km^2^ to end up total area of 82 km^2^ (12.2%), and water coverage continues to decrease by 10% or 67 km^2^ to result in the total area of 30 km^2^ (14.5%). Generally, cross-tabulation is used in this study to determine quantities of conversions from a particular Land Cover to another Land Cover category from a particular later date. The magnitudes of LULC class changes from agricultural land and water into open space and built-up area from 2001 to 2009 are tabulated in Table 3. In other words, the majority of the water body area is reduced and converted into open space and agricultural land, including certain areas that already transformed into built-up (Figures 3(a) and 3(b)). Meanwhile, Table 4 shows the LULC class changes from water and open space area into built-up area and agricultural land, as built-up areas are continuously increasing from open space and water coverage is transformed into agricultural land from 2009 to 2015 (Figures 3(b) and 3(c)).

### 5.2. Water Quality Assessment Based on Determination of Pollutant Sources

PCA was applied to compare composition patterns between water quality parameters and to determine the factors influenced by the identified regions in Malacca state. According to Table 6, there are 7 PCs identified through eigenvalues larger than 1 with 69% of total variance. Principal component (PC) 1 loadings with 15.3% of total variance have positive loadings for dissolved solids, electrical conductivity, and salinity, which are connected to agricultural activities and contribute to nonpoint source pollution through surface runoff [18]. Salinity pollution exists due to pesticide usage in oil palm and rubber plantations as well as animal husbandry (chickens, cows, and goats) carried out by some local residents along the Malacca River. Apart from that, erosion of riverbank due to dredging activity in the river is contributed to electrical conductivity pollution in the river. PC 2 explains positive loadings of turbidity and total suspended solid with total variance of 10.3%. This condition could happen when there are interruptions of human activities in terms of hydrologic modifications like dredging, water diversions, and channelization causing disruption in the Malacca River [16]. On the other hand, increasing population growth leading to land clearing increase for urban development [18, 19] and surface runoff cause road edge erosion [19] to happen within residential areas adjacent to the river. Next, PC 3 show positive loading on BOD and COD with the total of variance of 10.1%, which can be related to anthropogenic sources, having high possibility of coming from sewage treatment plant that contributed as point sources pollution [19].

PC 4 loadings with 10% of total variance have positive loadings on zinc and iron. Zinc pollution exists due to large numbers of houses and building development in urban and rural area that uses metallic roofs coated with zinc, where it can be mobilized into the atmosphere and waterways when contacting with acid rain or smog [19], while iron pollution happens because of agricultural activities in most parts of the rural area [18] and originating from industrial effluents in urban area [19]. PC 5 indicated positive loading of arsenic with total variance of 8.5%, showing that the pollutions are strong possibility of involving with the agricultural land [25]. PC 6 loadings with 8.0% of total variance have positive loadings on* E. coli* and total coliform, while negative loadings are dissolved oxygen. The presence of* E. coli* and total coliform pollution in the river is strongly connected with raw and municipal sewage from domestic and poultry farm mainly in rural and urban area. In addition to this, surface runoff and discharge from wastewater treatment plants from urban areas as well as dissolved oxygen pollution may be impacted by high levels of dissolved organic matter that consume large amounts of oxygen [19] and are suspected to come from agriculture activities and forest areas which are the dominant Land Use type in rural regions. Lastly, PC 7 resulted in positive loading of mercury with total variance of 6.8%, highly suspected to link with chemical industrial wastewater [25] that the majority occur at middle-stream and downstream of Malacca River. Therefore, the most likely sources of pollutants in terms of physicochemical and biological parameters are agriculture, residential activities, septic tank and sewage treatment plant activities, animal husbandry, industrial activities, and open space activities, which have an important role in specifying changes in LULC.

Continuously, CCA is carried out on the sets of data obtained from 7 PCs. There are 14 variables in the response data set, namely, biological parameter with* E. coli* and total coliform and physicochemical parameter including turbidity, DS, EC, salinity, DO, BOD, COD, TSS, As, Hg, Zn, and Fe (Table 7). Table 7 represents the results of CCA for biological and physicochemical variables. Correlation coefficients for canonical variates 1 and 2 were 0.841 and 0.660, respectively, indicating both are statistically significant (*p* < 0.000). The test statistic for canonical variates 1 and 2 is found to be *x*_2_^1^ = 620 with 24 degrees of freedom and *x*_2_^2^ = 311 with 11 degrees of freedom. This result indicates that both variates of 1 and 2 are having strong relationship with high correlation between the response and predictor sets of data; only variate 2 is higher than variate 1. The dominant variable in first canonical variate for biological variables (*U*_1_) is* E. coli*, while the dominant variables in *V*_1_ (physicochemical parameters) are DS, EC, DO, BOD, COD, Hg, and Zn. Next, the second canonical variates indicating the predictor variables are* E. coli* and total coliform, while the response variables have the result of turbidity, EC, salinity, TSS, As, and Fe. Considering the mentioned results, a regular pattern can be seen. From the first canonical variate it is indicated that residential and industrial activities have high percentage to cause pollutant sources, while second canonical variate indicates that agriculture, sewage treatment plant including septic tank, and animal husbandry activities proved to cause as pollutant sources and to react as nonpoint source pollution in the river.

**Figure 2:**
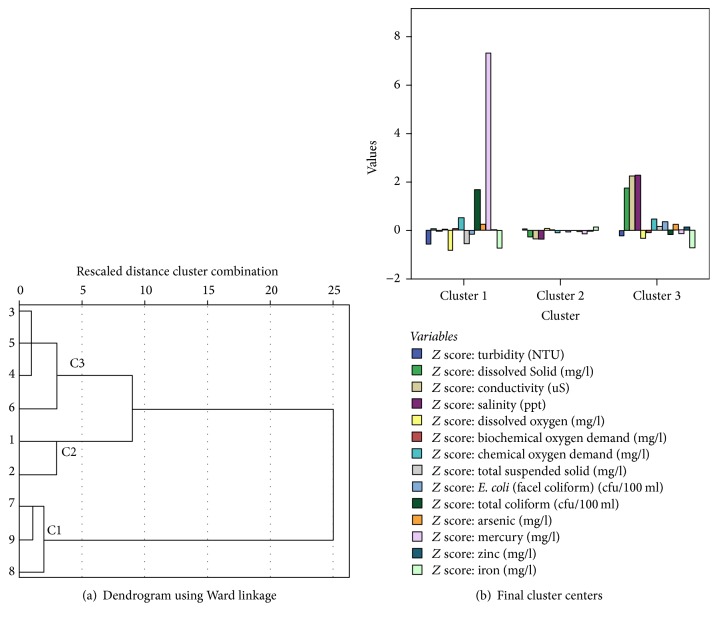
(a) Hierarchical cluster analysis using Ward's methods through Euclidean distance. (b) Nonhierarchical cluster analysis through *K*-means method.

**Figure 3:**
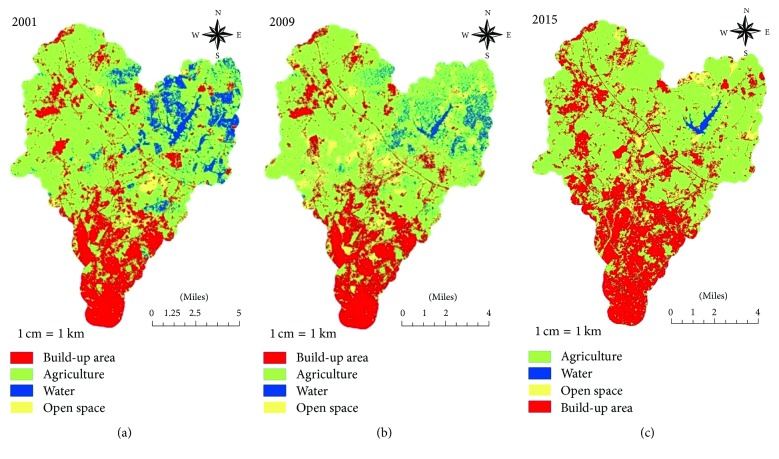
(a) Classified maps of Malacca River watershed in 2001. (b) Classified maps of Malacca River watershed in 2009. (c) Classified maps of Malacca River watershed in 2015.

Further analysis is carried out in hierarchical cluster analysis (HCA) and nonhierarchical cluster analysis (NHCA), as well as ANOVA between the LULC classes changes with river water quality data. The analysis of HCA using Ward's method indicates the results of three cluster areas, which can be divided into C1 with S7, S8, and S9; C2 with S1 and S2; and C3 with S3, S4, S5, and S6 (Figure 2(a)). The result provided will be further analyzed using nonhierarchical cluster analysis to obtain the correct classification of pollutant sources based on the PCA components in the location area involved. According to Table 8, NHCA confirmed four samples detected in cluster 1 with 275 cases involved to produce Hg, Fe, total coliform, and DO; cluster 2 has only 5 cases to produce two samples with salinity and EC; and cluster 3 detected three samples in 44 cases to produce salinity, EC, and DS. In other words, cluster 1 is significantly subjected to be involved with the industrial and residential activities, as well as sewage treatment plant [19], while cluster 3 is suspected to carry out agriculture, sewage treatment plant, and animal husbandry activities; and cluster 2 is involved with minor impact caused by agriculture and animal husbandry activities [18] (Figure 2(b)). Therefore, cluster 1 is likely to occur in urban area, cluster 3 is suburban area, and cluster 2 is rural area.

Lastly, as described in statistical analysis, analysis of variance (ANOVA) is carried out to obtain accurate result between LULC classes with river water quality of 15 years. Among the LULC classes, built-up areas are having the highest significance with 9 variables of water quality; vegetation is the second highest to have 8 variables significant with water quality; and the lowest significance is the open space with only 4 variables of water quality that resulted in ANOVA (Table 9). Built-up area is subjected to cause pollution in* E. coli*, total coliform, EC, BOD, COD, TSS, Hg, Zn, and Fe. In this condition, residential activities (BOD, COD,* E. coli*, total coliform, and Zn), industrial activities (Hg, Zn, and Fe), and sewage treatment plant (BOD, COD,* E. coli*, and total coliform) as well as animal husbandry (*E. coli*, total coliform) are suspected to become main pollutant sources to contaminate the Malacca River, as the majority occur in urban and suburban area. Meanwhile, vegetation area involves agriculture activities and forest land is suspected to cause pollution in river water quality. Agriculture activities with high usage of pesticide would cause salinization pollution; and high percentage of fertilizer would cause* E. coli*, total coliform, arsenic, and iron pollution. Indirectly, agriculture activities could disrupt the soil structure and cause EC as well as TSS in the river. These activities happen to result in nonpoint source pollution. Continuously, although DO is suspected to have contaminated in vegetation area, however, the variable is not considered due to no significance in analysis to provide the result of *F* (df = 2, *p* > 0.16) = 1.38. Probably minor cause of pollution from DO can be connected with forest land activities. Open space activities of LULC classes can be described as transition area for built-up area that converted from agriculture, as well as several areas from forest land into agriculture activities. On the other hand, hydrologic modification like dredging, water diversion, and channelization will cause erosion of riverbank to increase the pollution of turbidity, salinity, EC, and TSS.

## 6. Conclusion

Remote sensing is a tool to aid in detecting the magnitude of LULC change that has taken place in the Malacca River watershed for river water quality over the span of 15 years. It is divided into two parts: 2001 to 2009 for 9 years and 2009 to 2015 for 7 years. This research study has highlighted the application of remote sensing to develop LULC changes over time for the river water quality pollution based on pollutant sources. 7 PCs had been identified through PCA to result in DS, EC, salinity, turbidity, TSS, DO, BOD, COD, As, Hg, Zn, Fe,* E. coli*, and total coliform detected in the river water quality, which contribute possible detection of pollutant sources as agriculture activities, residential activities, industrial activities, septic tank, and sewage treatment plant activities, as well as animal husbandry activities. Simultaneously, selected variables from PCA will be applied into CCA to seek the relationship between the physicochemical parameters of response data and biological parameters of predictor data, with the result showing strong relationship and high correlation. The CCA indicate first canonical variate as* E. coli*, DS, EC, DO, BOD, COD, Hg, and Zn, to prove the existing of residential and industrial activities. Meanwhile, second canonical variate produces* E. coli*, total coliform, turbidity, EC, salinity, TSS, As, and Fe, which resulted as agriculture, sewage treatment plant as well as septic tank, and animal husbandry activities are carried out in the Malacca River watershed.

Afterwards, HCA is applied to determine possible area based on the pollution which occurred, indicating three clusters that consist of C1 with S7, S8, and S9; C2 with S1 and S2; and C3 with S3, S4, S5, and S6. Next, NHCA is used to obtain the correct classification of pollutant sources based on HCA cluster and PCA components, which defined that cluster 1 produces Hg, Fe, total coliform, and DO; cluster 2 produces salinity and EC; and cluster 3 produces salinity, EC, and DS. Overall, HCA and NHCA emphasize that cluster 1 occurs in urban area, cluster 3 is suburban area, and cluster 2 is rural area. Lastly, ANOVA between LULC and water quality data showed built-up area having contamination of* E. coli*, total coliform, EC, BOD, COD, TSS, Hg, Zn, and Fe, which highlighted the residential activities, industrial activities, and sewage treatment plant as well as animal husbandry that occur in urban and suburban area. Meanwhile, vegetation area of agriculture activities is suspected to cause EC, TSS, salinity,* E. coli*, total coliform, arsenic, and iron pollution, while forest land has minor impact to contaminate the river by DO pollution. Most of vegetation area occurs in suburban and rural area. Lastly, open space activities have pollution of turbidity, salinity, EC, and TSS due to hydrologic modification such as dredging, water diversion, and channelization. Overall, these research findings offer an effective solution to water quality management when large complex water quality data is involved, provided useful information in identifying pollution sources and understanding the river water quality with LULC change detection information providing references to policy maker in proper management of Land Use area.

## Figures and Tables

**Table 1:** Classes delineated on the basis of supervised classification.

Class name	Description
Vegetation	Including all agricultural and forest lands.
Built-up area	Including all residential, commercial, industrial, and transportation.
Water	Including all water bodies (river, lakes, gravels, stream, canals, and reservoirs).
Open space	Including all land areas that exposed soil and barren area influenced by human.

**Table 2:** Magnitude and percentage of LULC change from 2001–2009–2015.

Class	Total area and percentage						Magnitude of change			
	2001		2009		2015		2001–2009		2009–2015	
	Km^2^	%	Km^2^	%	Km^2^	%	Km^2^	%	Km^2^	%
Built-up area	196	29.3	245	36.6	337	50.3	+49	+7.3	+92	+13.7
Vegetation	271	40.4	202	30.1	221	33	−69	−10.3	+19	+2.9
Water	138	20.6	97	14.5	30	4.5	−41	−6.1	−67	−10
Open space	65	9.7	126	18.8	82	12.2	+61	+9.1	−44	−6.6
Total	670	100	670	100	670	100	0	0	0	0

**Table 3:** Cross-tabulation of LULC classes between 2001 and 2009 in (km^2^).

Class	Built-up area	Agriculture	Water	Open space	Total
Built-up area	196	27	22	0	245
Vegetation	0	136	66	0	202
Water	0	46	35	16	97
Open space	0	62	15	49	126
Total	196	271	138	65	**670**

**Table 4:** Cross-tabulation of LULC classes between 2009 and 2015 in (km^2^).

Class	Built-up area	Agriculture	Water	Open space	Total
Built-up area	245	62	12	18	337
Vegetation	0	140	40	41	221
Water	0	0	11	19	30
Open space	0	0	34	48	82
Total	245	202	97	126	**670**

**Table 5:** Mean (and standard deviation) values of water quality data along the Malacca River for years 2001, 2009, and 2015 (*n* = 20).

Category	Unit	Mean/SD								
		S1	S2	S3	S4	S5	S6	S7	S8	S9
pH	—	6.95 0.54	6.71 0.34	6.60 0.31	6.69 0.43	6.73 0.29	6.74 0.37	7.16 0.57	6.79 0.46	6.71 0.41
Temp	°C	28.16 1.39	28.39 1.62	28.10 1.49	27.83 1.50	27.69 1.33	27.72 1.27	28.47 1.10	29.12 1.70	28.43 1.09
Sal	%	15.58 10.62	3.22 4.84	1.38 2.65	0.19 0.33	0.04 0.03	0.04 0.02	15.89 11.69	0.22 0.19	0.04 0.03
EC	*µ*S/cm	19675.85 15404.51	669.08 804.15	751.91 1153.24	149.84 128.93	131.26 84.43	94.81 38.87	21440.05 17392.75	575.97 452.25	421.67 597.87
TSS	mg/l	87.22 98.09	50.44 55.81	92.31 60.05	137.89 127.04	172.25 176.29	168.11 156.76	103.11 98.85	38.25 14.56	110.61 99.65
DS	mg/l	11596.37 9874.23	496.24 547.70	367.15 488.24	136.13 168.28	53.87 23.44	55.05 22.50	6342.73 9143.48	741.55 2557.64	102.86 115.34
Tur	NTU	115.93 137.47	72.32 75.14	298.37 356.98	170.26 196.22	220.29 227.55	180.42 156.13	57.86 61.15	120.63 146.48	174.35 191.67
BOD	mg/l	5.36 3.42	7.14 3.49	4.83 2.37	3.94 1.82	3.97 1.59	4.11 1.45	5.61 1.86	8.64 1.82	4.69 2.35
COD	mg/l	40.86 23.79	39.53 19.64	31.72 12.46	24.56 12.60	26.83 11.46	21.94 4.93	33.86 16.44	41.69 16.47	25.36 8.36
DO	mg/l	3.03 1.61	3.17 1.54	3.94 1.74	5.26 1.32	5.82 1.11	6.04 0.95	4.21 1.25	4.35 2.43	5.96 0.74
NH_3_N	mg/l	1.91 1.95	2.64 1.68	1.54 1.11	0.42 0.33	0.32 0.27	0.38 0.33	1.95 1.86	3.64 2.17	0.48 0.26
As	mg/l	0.00 0.00	0.00 0.00	0.00 0.00	0.00 0.00	0.00 0.00	0.00 0.00	0.00 0.00	0.00 0.00	0.00 0.00
Hg	mg/l	0.00 0.00	0.00 0.00	0.00 0.00	0.00 0.00	0.00 0.00	0.00 0.00	0.00 0.00	0.00 0.00	0.00 0.00
Cd	mg/l	0.00 0.00	0.00 0.00	0.00 0.00	0.00 0.00	0.00 0.00	0.00 0.00	0.00 0.00	0.00 0.00	0.00 0.00
Cr	mg/l	0.00 0.00	0.00 0.01	0.00 0.00	0.00 0.00	0.00 0.00	0.00 0.00	0.00 0.00	0.00 0.00	0.00 0.00
Pb	mg/l	0.01 0.00	0.01 0.00	0.01 0.00	0.01 0.00	0.01 0.00	0.01 0.00	0.01 0.00	0.01 0.00	0.01 0.00
Zn	mg/l	0.04 0.03	0.03 0.02	0.04 0.03	0.04 0.03	0.04 0.03	0.04 0.03	0.04 0.02	0.04 0.02	0.04 0.03
Fe	mg/l	0.23 0.32	0.45 0.38	0.72 0.60	1.00 0.69	0.81 0.59	0.85 0.76	0.21 0.26	0.72 0.54	0.85 0.51
Total coliform	Count/100 ml	413219.44 489224.47	308180.56 253164.67	295641.67 394028.86	372263.89 665998.32	246772.22 331636.34	280902.78 304712.75	110611.11 106139.77	153483.33 89787.51	113047.22 87121.27
*E. coli*	Count/100 ml	73322.22 60836.76	45211.11 42416.73	26426.86 31735.22	11952.08 16765.82	8181.39 7885.95	31202.78 48253.61	14315.08 16147.08	31331.94 35706.19	22167.50 33138.80

Tur means turbidity; DS means dissolved solid; Con means electrical conductivity; Sal means salinity; Temp means temperature; DO means dissolved oxygen; BOD means biological oxygen demand; COD means chemical oxygen demand; TSS means total suspended solids; pH means acidic or basic water; NH_3_N means ammoniacal nitrogen; *E. coli* means *Escherichia* coliform; Coli means coliform; As means arsenic; Hg means mercury; Cd means cadmium; Cr means chromium; Pb means lead; Zn means zinc; Fe means iron; SD means standard deviation; S1 to S9 means Station 1 to Station 9.

**Table 6:** Varimax rotation PCs for water quality data within Malacca River basin.

Variables (unit)	Principle component						
	1	2	3	4	5	6	7
Turbidity (NTU)	−.084	**.761**	.020	.162	.154	−.087	−.040
Dissolved solid (mg/l)	**.806**	−.048	.016	−.087	.093	.111	−.021
Electrical conductivity (uS)	**.924**	.011	.045	−.034	−.120	.050	.003
Salinity (ppt)	**.913**	−.018	.010	−.014	.064	.031	.007
Temperature (°C)	.024	−.290	.318	−.370	−.525	.011	−.229
Dissolved oxygen (mg/l)	−.127	.254	−.207	−.184	.051	−**.636**	−.095
Biological oxygen demand (mg/l)	−.070	−.154	**.806**	−.074	.089	.053	−.014
Chemical oxygen demand (mg/l)	.233	.186	**.781**	.087	−.005	.083	.041
Total suspended solid (mg/l)	.056	**.816**	−.061	−.005	−.181	−.184	−.033
Acidity/alkalinity (pH)	.454	−.009	.198	−.396	−.546	−.084	−.023
Ammoniacal nitrogen (mg/l)	−.149	−.291	.549	−.275	−.124	.385	−.301
*E. coli* (cfu/100 ml)	.113	−.133	.076	.000	.105	**.679**	−.047
Coliform (cfu/100 ml)	−.001	−.188	−.019	.178	.500	**.602**	.497
Arsenic (mg/l)	.130	−.017	.217	−.124	**.763**	.048	−.155
Mercury (mg/l)	−.001	−.009	.068	−.065	−.064	−.013	**.870**
Chromium (mg/l)	−.079	.507	−.092	−.113	.008	.015	−.062
Zinc (mg/l)	.089	.080	.014	**.855**	.059	.056	.106
Iron (mg/l)	−.319	.023	−.056	**.746**	.018	−.008	−.173
Initial eigenvalue	3.297	2.797	2.357	2.061	1.856	1.821	1.535
% of variance	15.539	10.310	10.115	10.024	8.526	8.088	6.852
Cumulative %	15.539	25.849	35.964	45.987	54.514	62.602	69.455

^*∗*^The bold values are factor loadings above 0.6 that were taken after Varimax rotation is performed.

**Table 7:** Canonical correlation analysis of the data set.

Canonical variates	1	2
Canonical correlation	**0.841**	**0.660**
Chi-square	620	311
Degree of freedom	24	11
Significant level	0.000	0.000

*Biological parameter (unit)*		
*E. coli* (cfu/100 ml)	−**0.975**	−**0.276**
Total coliform (cfu/100 ml)	−0.118	**1.006**

*Physicochemical parameter (unit)*		
Turbidity (NTU)	0.100	**0.024**
Dissolved solid (mg/l)	−**0.212**	0.097
Electrical conductivity (uS)	**0.464**	−**0.527**
Salinity (ppt)	−0.404	**0.264**
Dissolved oxygen (mg/l)	**0.724**	0.311
Biochemical oxygen demand (mg/l)	−**0.211**	0.037
Chemical oxygen demand (mg/l)	−**0.128**	−0.092
Total suspended solid (mg/l)	0.118	−**0.377**
Arsenic (mg/l)	−0.016	**0.246**
Mercury (mg/l)	**0.176**	0.518
Zinc (mg/l)	−**0.173**	0.623
Iron (mg/l)	0.197	−**0.098**

**Table 8:** The physicochemical and biological properties classified by the *K*-mean method.

Variable (unit)	Frequency	Cluster 1	Cluster 2	Cluster 3
Turbidity (NTU)	Mean range	−0.63 −0.71~−0.55	1.56 3.07~0.05	−0.4 −0.56~−0.24
Dissolved solid (mg/l)	Mean range	0.36 0.66~0.06	−0.33 −0.37~−0.28	0.71 −0.32~1.73
Electrical conductivity (uS)	Mean range	0.26 0.55~−0.03	−0.39 −0.42~−0.36	0.92 −0.39~2.23
Salinity (ppt)	Mean range	0.13 0.21~0.04	−0.42 −0.48~−0.36	0.93 −0.41~2.26
Dissolved oxygen (mg/l)	Mean range	−0.77 −0.71~−0.83	0.49 0.91~0.07	−0.22 −0.11~−0.33
Biochemical oxygen demand (mg/l)	Mean range	0.16 0.23~0.08	−0.06 −0.13~0.01	−0.11 −0.13~−0.08
Chemical oxygen demand (mg/l)	Mean range	0.54 0.56~0.52	0.27 0.62~−0.08	−0.53 −1.51~0.46
Total suspended solid (mg/l)	Mean range	−0.66 −0.77~−0.55	3.54 7.09~−0.01	−0.09 −0.32~0.15
Arsenic (mg/l)	Mean range	0.32 0.37~0.27	−0.33 −0.61~−0.04	0.06 −0.12~0.24
Mercury (mg/l)	Mean range	8.67 10.02~7.31	−0.13 −0.15~−0.11	−0.13 −0.15~−0.11
Zinc (mg/l)	Mean range	−0.51 −1.04~0.02	−0.53 −1.04~−0.02	0.49 0.85~0.12
Iron (mg/l)	Mean range	−0.9 −1.06~−0.74	−0.22 −0.58~0.13	0.79 2.29~−0.72
*E. coli* (cfu/100 ml)	Mean range	−0.37 −0.58~−0.16	−0.28 −0.51~−0.05	−0.07 −0.48~0.35
Total coliform (cfu/100 ml)	Mean range	0.62 −0.44~1.68	−0.32 −0.63~−0.01	4.33 8.81~−0.16

Number of samples		4	2	3
Sampling stations		7, 8, 9	1, 2	3, 4, 5, 6

**Table 9:** ANOVA between LULC classes changes with water quality in 2001, 2009, and 2015.

Parameter (unit)	LULC classes (ha)			ANOVA results		
	Built-up area	Vegetation	Open space	DF	*F*	*p* value
*E. coli* (cfu/100 ml)	**181.14**	**88.22**	45.00	2	13.43	0.0001
Total coliform (cfu/100 ml)	**219.63**	**80.71**	55.53	2	14.48	0.0000
Turbidity (NTU)	64.35	28.75	**7.10**	2	9.77	0.0001
Dissolved solid (mg/l)	111.46	59.81	12.77	2	2.65	0.0773
Electrical conductivity (uS)	**78.71**	**22.57**	**6.01**	2	7.54	0.0001
Salinity (ppt)	1.773	**0.891**	**0.221**	2	2.33	0.0243
Dissolved oxygen (mg/l)	15.72	**8.61**	2.22	2	1.38	0.1634
Biochemical oxygen demand (mg/l)	**21.34**	19.18	7.46	2	11.71	0.0000
Chemical oxygen demand (mg/l)	**68.47**	32.21	18.59	2	9.63	0.0001
Total suspended solid (mg/l)	**54.98**	**47.33**	**12.76**	2	6.66	0.0001
Arsenic (mg/l)	0.0015	**0.0007**	0.0005	2	19.27	0.0000
Mercury (mg/l)	**0.0017**	0.0013	0.0007	2	25.85	0.0000
Zinc (mg/l)	**0.0156**	0.0120	0.0090	2	4.26	0.0178
Iron (mg/l)	**0.34**	**0.18**	0.12	2	10.16	0.0000

^*∗*^One-way ANOVA with *p* < 0.05 is significant.
